# Factors Associated with Virological Non-suppression among HIV-Positive Patients on Antiretroviral Therapy in Uganda, August 2014–July 2015

**DOI:** 10.1186/s12879-017-2428-3

**Published:** 2017-05-03

**Authors:** Lilian Bulage, Isaac Ssewanyana, Victoria Nankabirwa, Fred Nsubuga, Christine Kihembo, Gerald Pande, Alex R. Ario, Joseph KB Matovu, Rhoda K. Wanyenze, Charles Kiyaga

**Affiliations:** 1 Uganda Public Health Fellowship Program – Field Epidemiology Track, Kampala, Uganda; 2 Central Public Health Laboratories, Ministry of Health, Kampala, Uganda; 3 Makerere University School of Public Health, Kampala, Uganda

**Keywords:** Antiretroviral therapy, Virological non-suppression, Uganda

## Abstract

**Background:**

Despite the growing number of people on antiretroviral therapy (ART), there is limited information about virological non-suppression and its determinants among HIV-positive (HIV+) individuals enrolled in HIV care in many resource-limited settings. We estimated the proportion of virologically non-suppressed patients, and identified the factors associated with virological non-suppression.

**Methods:**

We conducted a descriptive cross-sectional study using routinely collected program data from viral load (VL) samples collected across the country for testing at the Central Public Health Laboratories (CPHL) in Uganda. Data were generated between August 2014 and July 2015. We extracted data on socio-demographic, clinical and VL testing results. We defined virological non-suppression as having ≥1000 copies of viral RNA/ml of blood for plasma or ≥5000 copies of viral RNA/ml of blood for dry blood spots. We used logistic regression to identify factors associated with virological non-suppression.

**Results:**

The study was composed of 100,678 patients; of these, 94,766(94%) were for routine monitoring, 3492(4%) were suspected treatment failures while 1436(1%) were repeat testers after suspected failure. The overall proportion of non-suppression was 11%. Patients on routine monitoring registered the lowest (10%) proportion of non-suppressed patients. Virological non-suppression was higher among suspected treatment failures (29%) and repeat testers after suspected failure (50%). Repeat testers after suspected failure were six times more likely to have virological non-suppression (OR_adj_ = 6.3, 95%CI = 5.5–7.2) when compared with suspected treatment failures (OR_adj_ = 3.3, 95%CI = 3.0–3.6). The odds of virological non-suppression decreased with increasing age, with children aged 0–4 years (OR_adj_ = 5.3, 95%CI = 4.6–6.1) and young adolescents (OR_adj_ = 4.1, 95%CI = 3.7–4.6) registering the highest odds. Poor adherence (OR_adj_ = 3.4, 95%CI = 2.9–3.9) and having active TB (OR_adj_ = 1.9, 95%CI = 1.6–2.4) increased the odds of virological non-suppression. However, being on second/third line regimens (OR_adj_ = 0.86, 95%CI = 0.78–0.95) protected patients against virological non-suppression.

**Conclusion:**

Young age, poor adherence and having active TB increased the odds of virological non-suppression while second/third line ART regimens were protective against non-suppression. We recommend close follow up and intensified targeted adherence support for repeat testers after suspected failure, children and adolescents.

## Background

The number of people accessing antiretroviral therapy (ART) has gradually increased in the recent years [1]. With the rising numbers of patients accessing ART, it is important to sustain treatment success and limit development of treatment failure. To allow timely detection of treatment failures, WHO in July 2013 recommended use of viral load testing as the gold standard to monitor patients response to ART [2]. Establishment of the virological suppression status among patients enrolled on ART is important for timely detection of treatment failures, identification of patients in need of more intensive adherence support and minimizes development of drug resistance and unnecessary switch to expensive and limited ART regimen options [3, 4].

The Ugandan guidelines recommend that viral load testing should be done 6 months after initiating ART and thereafter annually for people who have achieved viral suppression. However, people with detectable viral loads undergo targeted intensified adherence support for 6 months followed by confirmatory viral load testing in order to differentiate poor adherence from treatment failure. Those with treatment failure as defined by two detectable viral load measurements above the threshold are switched to second-line ART [2].

Previous studies have highlighted a number of factors that may be associated with virological suppression. Patients with WHO clinical staging 4 are more likely to be non-suppressed while those whose health status is evaluated by physicians on each clinic visit are less likely to experience virological failure [5]. Children and adolescents on ART are more likely to have high viral loads [6]. Suboptimal adherence, poor tolerability, and drug and food interactions, CD4 cell count, treatment history and drug-resistance (primary or transmitted) have also been associated with virological non-suppression. Suboptimal adherence and drug intolerance are the major cause of regimen discontinuations and hence virological failure [7–9]. Virological non-suppression may also be caused by patient-related factors such as co-morbidities, incomplete medication adherence and missed clinic appointment and interruption of or intermittent access to ART, and ARV regimen related factors such as drug adverse effects, suboptimal pharmacokinetics, suboptimal virological potency and food requirements, amongst other factors [10].

In 2014 the Joint United Nations Programme on HIV/AIDS (UNAIDS) set new targets towards elimination of HIV, including diagnosis of 90% of HIV infected individuals, access to treatment for 90% of identified HIV infected persons, and 90% viral suppression among those initiated on treatment [11]. These targets have since been adopted by several countries including Uganda. Thus, viral load is a critical indicator for HIV treatment quality and tracking of progress towards national and global indicators.

Despite the increasing number of HIV+ patients accessing ART [1], there is limited information about non-suppression rates amongst the different groups of people enrolled in care in Uganda and many resource limited settings in general. Studies that have highlighted the factors associated with virological suppression in most developed countries and resource limited settings have used lower cut-offs to determine non-suppression. The thresholds used range from 300 to 500 copies/ml of blood [12]. However, since several clinical and epidemiological studies have highlighted the risk of HIV transmission being very low when the viral load is lower than 1000 copies/ml, WHO recommends using 1000 copies/ml as the threshold when using plasma and 5000 copies/ml for dry blood spots (DBS) [2, 13]. Uganda and other countries such as Swaziland are using the WHO recommended thresholds. Most Ugandan studies that have assessed viral suppression have been based on small numbers [14, 15]. Finally, the relative contribution of the factors leading to virological non-suppression may vary across settings and population groups and context specific data are critical to the implementation of corrective measures. The aim of this study was to estimate the proportion of virologically non-suppressed HIV+ patients who had been on ART for at least 6 months and identify factors associated with virological non-suppression using a large national dataset of routinely collected program data at the Central Public Health Laboratories (CPHL) in Uganda between August 2014 and July 2015.

## Methods

### Study design

This was a descriptive cross-sectional study using a large national dataset of routinely collected program data (sample size 100,678) that were submitted to the Central Public Health Laboratories (CPHL) from all health facilities in Uganda between August 2014 and July 2015.

### Study setting

The study was conducted at CPHL where the centralized VL testing is done in Uganda. Centralized monitoring of response to ART using viral load as a gold standard started in August 2014. The samples come from all over the country from both private and government health facilities offering HIV ART services. Uganda is located within the East African region with a population of 34.9 million [16] and a general HIV prevalence of 7.3% [17]. According to the 2014 Uganda HIV and AIDS Country Progress Report, 1,500,000 people were living with HIV in 2014 [18]. Of these, 750,896 (50% coverage) were active on ART by Dec 2014 [18]. There were 125,705 new active patients on ART by end of 2014 [18]. According to the viral load testing request form version for Uganda, the three main reasons for viral load testing are: 1) routine testing; 2) suspected failure, and 3) repeat viral load testing after suspected treatment failure for patients who were virologically non-suppressed on first time testing and underwent enhanced adherence support for 6 months and submitted a follow-up sample at the end of 6 months.

### Study participants and data collection

We used VL testing data for samples corresponding to HIV positive patients who had been on ART for at least 6 months. Data generated from the start of VL testing at CPHL in August 2014 to July 2015 were considered for the study.

### Measures

The primary outcome was virological non-suppression, defined as having ≥1000 copies of viral RNA/ml of blood for plasma or ≥5000 copies of viral RNA/ml of blood for dry blood spots [19]. We abstracted data on viral load testing results (for plasma and DBS) measured in terms of viral RNA copies/ml of blood, age, sex, duration of treatment, treatment line (first, second, third), identification for treatment initiation at baseline (ePMTCT option B+, child under 15, CD4 < 500 and TB infection), pregnancy status, breast feeding status, having active TB, health facility level, reasons for viral load testing (routine monitoring, suspected treatment failure, and repeat VL test after suspected treatment failure [for patients who were not suppressed on first time testing underwent enhanced adherence support for six months and submitted a follow up at the end of the six months]).

### Data analysis

We analyzed the entire study data set to estimate the proportion of patients with virological non-suppression, and to identify factors associated with virological non-suppression. In-addition, we conducted sub-group analyses in line with the 3 major reasons for viral load testing in Uganda (routine monitoring of response to treatment, suspected treatment failure, and repeat viral load testing after suspected failure). We used univariate analysis to establish the socio-demographic and clinical characteristics of the study population, the overall and group specific proportions of patients with virological non-suppression. Virological non-suppression proportion was defined as the percentage of the number of total not suppressed among the total number tested. The proportion with viorological non-suppression were further evaluated by age group, gender, duration on treatment, reported adherence levels, pregnancy status, breast feeding status, having active TB, treatment line, reason for VL test (routine monitoring, suspected treatment failure, and repeat VL test after suspected failure) and health facility level.

We created the outcome variable of suppression status by categorizing the viral load results into two groups. All results <1000 or 5000 copies/ml of blood for plasma and DBS respectively were categorized as suppressed while ≥1000 or 5000 copies/ml of blood for plasma and DBS respectively were categorized as not suppressed. At the exploratory analysis stage, we realized that the percentage of variables with missing values was <10% of all the variables in the data set. Missing values were dropped automatically from each variable and analysis was conducted based on the totals with complete records.

We used bivariate analysis to determine strengths of association between the independent variables and the outcome variable (virological suppression status). Crude odds ratios (OR) and 95% confidence intervals were calculated. We used multivariate logistic regression to identify factors independently associated with virological non- suppression. Adjusted odds ratios (OR) and 95% confidence intervals were calculated. We considered the reason for viral load testing (routine monitoring, suspected treatment failures, and repeat testers after suspected treatment failure) in Uganda as the main exposure variable of interest. We adjusted for age, sex, ARV adherence, treatment line (first, second/third), identification for treatment initiation at baseline (ePMTCT option B+, child under 15, CD4 < 500 and TB infection), having active TB, duration on treatment, and health facility level. We dropped the option “other” under the option for indication for treatment initiation at baseline when conducting bivariate and multivariate analysis. This was due to the fact that it was not clear which categories of patients were categorized as “other”. In the sub-analyses, age group was considered to be the main exposure variable of interest. In addition, factors were entered into logistic regression models based on biological plausibility, previous literature, and statistical significance in bivariate analysis. We tested for interaction and did not find any statistically significant interaction terms to report on. Pregnant and breast feeding status were excluded from model building because they only apply to females. Statistical significance was considered at *p*-value <0.05 (two-sided). Data were entered into Excel 2007 and analyzed using Epi-info version 7.

### Ethical consideration

We used program data collected for routine patient care at all health facilities in Uganda and submitted to the Central Public Health Laboratories (CPHL) which is mandated to conduct centralized viral load testing in Uganda. All data did not carry personal identifiers. The data were not accessible by any other third parties other than the study team. Permission to use the data was sought from the Ugandan Ministry of Health under whose mandate CPHL falls.

## Results

### Patients’ characteristics

Table 1 shows the characteristics of the 100,678 patients whose viral load data were available for analysis. Of these, 94,766 (94%) were for routine monitoring, 3492 (4%) were for suspected treatment failures while 1436 (1%) were for repeat testers after suspected failure. In general, females contributed the biggest proportion of the study sample, ranging from 55 to 68% across the three patient-subcategories. Most of the patients were aged 35+ years: 53,489 (56%) among patients for routine monitoring, 2140 (61%) among suspected treatment failures and 430(30%) among repeat testers after suspected failure. Majority of the patients had been initiated on ART basing on their CD4 < 500, i.e., 70,116 (76%) among patients for routine monitoring, 2832 (85%) among suspected treatment failures and 839 (62%) among repeat testers after suspected failure. All the 3 categories had majority of the patients on first line regimens, i.e., routine monitoring 89,382 (95%), suspected treatment failures 3177 (92%) and repeat testers after suspected failure 1070 (75%). Reported adherence (>95%) was highest among patients for routine monitoring 81,478 (89%), followed by repeat testers after suspected failure 1005 (74%), and lowest among suspected treatment failures 2278 (68%). The proportion of women who were either pregnant or breast feeding ranged from 1 to 5% across all the 3 patient categories. Patients with active TB represented only 1–5% of the study sample across all the patient categories.

**Table 1:** Socio-demographic and clinical characteristics of patients, August 2014–July 2015 (*N* = 100,678, routine monitoring, *n* = 94,766, suspected treatment failures, *n* = 3492 and second time testers, *n* = 1436)

Variable	General-*N*(%)	Routine monitoring, *n*(%)	Suspected treatment failures, *n*(%)	Repeat testers after suspected failure, *n*(%)
Age group				
0–4	1763(2.0)	1637(1.7)	45(1.3)	65(4.5)
5–9	3983(4.0)	3681(3.9)	111(3.2)	172(11.9)
10–14	4231(4.0)	3898(4.1)	136(3.9)	168(11.6)
15–19	3085(3.0)	2689(2.8)	189(5.4)	180(12.7)
20–24	4781(5.0)	4436(4.7)	176(5.0)	131(9.1)
25–29	10,695(11.0)	10,192(10.8)	257(7.4)	131(9.2)
30–34	15,494(15.0)	14,744(15.6)	438(12.5)	159(11.0)
35+	56,646(56.0)	53,489(56.4)	2140(61.3)	430(29.9)
Sex				
Male	32,203(32.0)	29,873(32.0)	1539(45.0)	515(36.0)
Female	67,097(68.0)	63,598(68.0)	1918(55.0)	900(64.0)
Duration on treatment				
0–2 Years	36,341(36.0)	3,4610(36.6)	1002(28.8)	298(20.8)
3–5 Years	32,928(33.0)	30,876(32.7)	1240(35.7)	548(38.3)
6–10 Years	28,423(28.0)	26,533(28.1)	1110(31.9)	534(37.3)
> 10 Years	2726(3.0)	2518(2.7)	125(3.6)	52(3.6)
Indication for treatment initiation				
TB infection	4076(4.0)	3721(4.0)	129(3.9)	127(9.4)
Other	1496(2.0)	1392(2.0)	72(2.2)	24(1.8)
Child under 15	9820 (10.0)	9550(10.0)	83(2.5)	59(4.4)
PMTCT/Option B+	7712(8.0)	7117(8.0)	234(7.0)	300(22.4)
CD4 < 500	74,401(76.0)	70,116(76.0)	2832(84.5)	839(62.0)
Report ARV adherence				
< 85%	1292(1.3)	964(1.0)	253(7.5)	62(5.0)
Fair 85–95%	10,065(10.4)	8798(10.0)	832(24.7)	291(21.0)
Good >95%	85,440(88.3)	81,478(89.0)	2278(67.7)	1005(74.0)
Treatment line				
First line	94,476(94.45)	89,382(95.0)	3177(92.2)	1070(75.0)
Second line	5497(5.5)	4815(5.0)	264(7.7)	354(25.0)
Third line	50(0.05)	39(0.0)	4(0.1)	4(0.0)
Pregnant				
Yes	2009(3.0)	1950(2.0)	27(1.0)	33(2.4)
Breast feeding				
Yes	4697(7.0)	4647(5.0)	52(2.0)	35(3.0)
Active TB				
Yes	668(1.0)	575(0.6)	77(2.3)	15(1.1)
Level of health facility				
HCII + HCIII	1,5404(15.0)	14,757(16.0)	376(11.0)	85(6.0)
HCIV + General hospital	26,102(26.0)	24,713(26.0)	969(28.0)	155(11.0)
Specialized HIV cares services	13,944(14.0)	13,224(14.0)	398(11.0)	146(10.0)
RRH + NRH	45,203(45.0)	42,047(44.0)	1747(50.0)	1050(73.0)

Denominator for pregnant/breastfeeding mothers was total females in the sample.

*HCII* health centre two, *HCIII* health centre three, *HCIV* health centre four, *RRH* regional referral hospitals, *NRH* national referral hospitals

### Proportion of patients with virological non-suppression

Table 2 shows the proportion of patients with overall virological non-suppression and by the three patient sub-categories. The overall proportion of virologically non-suppressed patients in the study sample was 11%. In general, repeat testers after suspected failure had the highest proportion of patients with virological non-suppression (50%), followed by suspected treatment failures (29%) and least routine monitoring patients (10%). In-addition, the proportion of virologically non-suppressed patients were highest among the repeat testers after suspected failure, followed by suspected treatment failures and lastly patients for routine monitoring considering all the variables explored.

**Table 2:** Proportion of HIV patients on ART with virological non-suppression, (*N* = 100,678, routine monitoring, *n* = 94,766, suspected treatment failures, *n* = 3492 and second time testers, *n* = 1436)

Variable	Non-suppression status, *n*(%)			
	General	Routine monitoring	Suspected treatment failures	Repeater testers after suspected failure
Overall	10,805 (11)	8966(10)	1018(29)	716(50)
Sex				
Female	6524(10)	5437(9)	592(31)	427(47)
Male	4132(13)	3402(11)	416(27)	278(54)
Age category				
0–4	507(29)	444(27)	22(49)	34(52)
5–9	916(23)	754(21)	55(50)	104(60)
10–14	960(23)	765(20)	83(61)	105(63)
15–19	837(27)	614(23)	102(54)	118(66)
20–24	746(16)	595(13)	81(46)	65(50)
25–29	1141(11)	945(9)	117(46)	65(50)
30–34	1579(10)	1337(9)	154(35)	70(44)
35+	4119(7)	3512(7)	404(19)	155(36)
Breast feeding				
Yes	297(6)	262(6)	22(42)	14(40)
No	5964(10)	8420(10)	956(29)	679(50)
Pregnant				
Yes	153(8)	128(7)	13(48)	14(42)
No	6113(10)	8554(10)	966(29)	681(50)
Active TB				
Yes	131(20)	85(15)	34(44)	12(80)
No	10,236(11)	8542(9)	932(29)	684(50)
Treatment line				
First	9945(11)	8351(9)	925(29)	582(54)
Second	736(13)	531(11)	75(28)	128(36)
Third	11(22)	4(10)	3(75)	3(75)
Duration on treatment				
0–2 Years	3680(10)	3198(9)	291(29)	133(45)
3–5 Years	3900(12)	3226(10)	386(31)	264(48)
6–10 Years	2928(10)	2304(9)	309(28)	294(55)
> 10 Years	257(9)	210(8)	24(19)	22(42)
Indication for treatment initiation				
TB infection	194(13)	159(11)	19(26)	(16(67)
Other	604(15)	466(13)	49(38)	77(61)
Child under 15	1826 (24)	1508 (21)	135(58)	170(57)
PMTCT/Option B+	589(6)	549(6)	18(22)	12(20)
CD4 < 500	7169(10)	5955(9)	750(26)	401(48)
Reported adherence level				
< 85%	453(35)	324(34)	85(34)	38(61)
85–94%	1659(17)	1206(14)	269(32)	168(58)
> 95%	8145(10)	6998(9)	622(27)	465(46)
Level of health facility				
HCII + HCIII	1455(9)	1295(9)	104(28)	36(42)
HC IV + General hospital	2293(9)	1994(8)	228(24)	39(25)
Specialized HIV care services	1482(11)	1242(9)	148(37)	83(57)
RRH + NRH	5575(12)	4435(11)	538(31)	558(53)

Denominator for pregnant/breastfeeding mothers was total females in the sample

*HCII* health centre two, *HCIII* health centre three, *HCIV* health centre four, *RRH* regional referral hospitals, *NRH* national referral hospitals

In general, the proportion of virologically non-suppressed patients decreased with increasing age, with the highest proportion being 29% for children aged 0–4 years, followed by 27% for adolescents aged 15–19 years and least 7% for adults aged 35+ years. The same trend is observed across the 3 patient sub-categories. Males (13%) had a slightly higher proportion of non-suppressed patients compared the females (10%). Breast feeding (6%) and pregnant (8%) mothers had the lowest proportion of virologically non-suppressed patients amongst all the different patient categories explored. In general, virological non-suppression was highest amongst patients with reported adherence levels of <85% (35%) followed by patients with 85–94% (17%) and least among those with adherence levels >95% (10%). Regional and National referral hospitals had the highest proportion of virologically non-suppressed patients (12%), followed by specialized HIV care services with 11%, and least health centre IV and general hospitals, and health centre II and III each with 9% (Table 2).

### Factors associated with virological non-suppression

Table 3 shows the crude and adjusted odds ratios associated with virological non-suppression by reason for viral load testing and other background characteristics. At bivariate analysis level, the reason for viral load testing was significantly associated with virological non-suppression: Suspected treatment failures (OR_crude_ = 4.2, 95%CI = 3.6–4.2) and repeat testers after suspected failure (OR_crude_ = 9.5, 95%CI = 8.6–10) were more likely to be virologically non-suppressed, compared with routine testers. In addition, young age was significantly associated with virological non-suppression: Children aged 0–4 years (OR_crude_ = 5.1, 95%CI = 4.6–5.7) and adolescents aged 15–19 years (OR_crude_ = 4.7, 95%CI = 4.4.4–5.2) were more likely to be virologically non-suppressed compared with patients aged 35+ years. Patients with reported adherence level < 85% (OR_crude_ = 5.1, 95%CI = 4.6.4–5.8) were also more likely to be virologically non-suppressed compared to patients with >95% adherence level. Similarly, patients on second/third line treatment (OR_crude_ = 1.3, 95%CI = 1.3.4–1.4), patients with active TB (OR_crude_ = 2.1, 95%CI = 1.7–2.5) were also more likely to be virologically non-suppressed. Pregnancy (OR_crude_ = 0.77, 95%CI = 0.65–0.91) and breast feeding (OR_crude_ = 0.61, 95%CI = 0.54–0.69) were protective against virological non-suppression (Table 3).

**Table 3:** Factors associated with virological non-suppression among HIV patients on ART, August 2014–July 2015, (*N* = 100,678)

Variable	Not suppressed (%)	Suppressed (%)	OR_crude_(95%CI)	OR_adj_(95%CI)^a^
Reason for testing				
Routine testing	8966(84)	8966(96)	1.0	1.0
Suspected treatment failure	1018(10)	2474(3.0)	4.2(3.6–4.2)	3.3(3.0–3.6)
Repeat testers after suspected failure	716(6)	720(1.0)	9.5(8.6–10)	6.3(5.5–7.2)
Age category				
35+	4119(38)	52,527(59)	1.0	1.0
34–30	1579(15)	13,915(16)	1.4(1.4–1.5)	1.6(1.5–1.7)
29–25	1141(11)	9554(11)	1.5(1.4–1.6)	1.9(1.7–2.0)
24–20	746(7)	4035(5)	2.4(2.2–2.6)	2.7(2.5–3.0)
19–15	837(8)	2248(3)	4.7(4.4–5.2)	4.1(3.7–4.6)
14–10	960(9)	3271(4)	3.7(3.5–4.1)	3.3(2.9–3.7)
9–5	916(9)	3067(3)	3.8(3.5–4.1)	3.4(3.1–3.8)
4–0	507(5)	1256(1)	5.1(4.6–5.7)	5.3(4.6–6.1)
Indication for treatment initiation				
PMTCT/Option B+	589(6)	9231(11)	1.0	1.0
Child <15 Years	1826(19)	5886(7)	4.9(4.4–5.6)	2.1(1.9–2.4)
TB infection	194(2)	1302(2)	2.3(1.9–2.8)	2.1(1.8–2.6)
CD4 < 500	7169(73)	67,232(80)	1.7(1.5–1.8)	2.0(1.8–2.2)
ARV adherence				
> 95%	8145(79)	77,295(89)	1.0	1.0
85–94%	1659(17)	8406(8)	1.9(1.8–2.0)	1.6(1.5–1.7)
< 85%	453(4)	839(1)	5.1(4.6–5.8)	3.4(2.9–3.9)
Treatment line				
First line	9945(93)	8453(95)	1.0	1.0
Second/Third line	747(7)	4800(5)	1.3(1.3–1.4)	0.86(0.78–0.95)
Sex				
Female	6524(61)	60,573(68)	1.0	1.0
Male	4132(39)	28,071(32)	1.4(1.3–1.4)	1.2(1.1–1.3)
Active TB				
No	1,0236(99)	86,650(99)	1.0	1.0
Yes	131(1)	537(1)	2.1(1.7–2.5)	1.9(1.6–2.4)
Health Facility Level				
HCII + HCIII	1455(13)	13,949(16)	1.0	1.0
HCIV + General hospital	2293(21)	23,809(27)	0.92(0.86–0.99)	0.89(0.82–0.97)
Specialized HIV services	1482(14)	12,462(14)	1.1(1.1–1.2)	1.2(1.1–1.3)
RRH + NRH	5575(52)	39,628(44)	1.3(1.3–1.4)	1.0(0.96–1.1)
Duration on treatment				
0–2 Years	3680(34)	32,661(36)	1.0	1.0
3–5 Years	3900(36)	29,028(32)	1.2(1.1–1.3)	1.3(1.2–1.4)
6–10 Years	2928(27)	2549(28)	1.0(0.97–1.1)	1.1(1.1–1.2)
> 10 Years	257(3)	2469(4)	0.92(0.81–1.1)	0.91(0.78–1.1)
Breast feeding				
No	5964(95)	54,369(93)	1.0	
Yes	297(5)	4400(8)	0.61(0.54–0.69)	−
Pregnant				
No	6113(98)	56,841(97)		
Yes	153(2)	1856(3)	0.77(0.65–0.91)	−

Denominator for pregnant/breastfeeding mothers was total females in the sample

*HCII* health centre two, *HCIII* health centre three, *HCIV* health centre four, *RRH* regional referral hospitals, *NRH* national referral hospitals

^a^We adjusted for age, sex, ARV adherence, treatment line (first, second/third), identification for treatment initiation at baseline (ePMTCT option B+, child under 15, CD4 < 500 and TB infection), having active TB, duration on treatment, and health facility level

At multivariate analysis level, being a suspected treatment failure (OR_adj_ = 3.3, 95%CI = 3.0–3.6), and repeat tester after suspected failure (OR_adj_ = 6.3, 95%CI = 5.5–7.2), being a child aged 0–4 years (OR_adj_ = 5.3, 95%CI = 4.6–6.1), being an adolescent aged 15–19 years (OR_adj_ = 4.1, 95%CI = 3.7–4.6), having reported adherence level < 85% (OR_adj_ = 3.4, 95%CI = 2.9.4–3.9), and having active (OR_adj_ = 1.9, 95%CI = 1.6–2.4) were significantly associated with increased odds of virological non-suppression. However, being on second/third line treatment (OR_adj_ = 0.86, 95%CI = 0.78–0.95) reduced the odds of virological non-suppression (Table 3).

### Factors associated with virological non-suppression: Sub-group analysis

In a sub-group analysis of the factors associated with virological non-suppression among suspected treatment failures and repeat testers after suspected failure, we found that, among suspected treatment failures, breast feeding (OR_crude_ = 1.8, 95%CI = 1.0–3.2) and pregnancy (OR_crude_ = 2.3, 95%CI = 1.1–4.9) increased the odds of being virologically non-suppressed (at bivariate analysis level). On the other hand, among repeat testers after suspected failure, having active TB was associated with the highest odds of virological non-suppression (OR_adj_ = 7.0, 95%CI = 1.4–32.1).

## Discussion

In this descriptive cross-sectional study conducted to estimate the proportion of patients virologically non-suppressed and to identify the factors associated with virological non-suppression, we found an overall proportion of non-suppression of 11%. Amongst the 3 sub-patient categories, patients on routine monitoring registered the lowest while repeat testers after suspected failure registered the highest proportion of non-suppressed patients. Being a suspected treatment failure, repeat tester after suspected treatment failure, young age, poor adherence and having active TB increased the odds of virological non-suppression. Being on second/third line treatment decreased the odds of virological non-suppression. Repeater testers after suspected failure with active TB registered the highest odds of non-suppression compared to TB patients amongst other sub-categories.

Although our study reveals the overall proportion of non-suppressed patients being relatively low, we observe sub-groups such as children and adolescents, patients with adherence level < 85%, patients with active TB, and repeat testers after suspected failure having relatively higher virological non-suppression levels. This calls for MOH to enhance efforts in the identified sub-groups to improve virological suppression.

Our findings show that repeat testers after suspected failure registered the highest non-suppression. Although the Uganda viral load monitoring guidelines recommend enhanced adherence counseling and support for patients whose first VL result is beyond the threshold, it is not clear whether the health care system is giving enough attention to these patients. The Uganda guidelines recommend use of text messages for adherence fostering. Other countries like Swaziland have developed and implemented targeted adherence support tools which recommend more frequent clinic reviews (1-monthly drug pick-ups from 3-monthly pick-ups), stepped-up adherence counseling (3 sessions) and education on the value for VL testing among other interventions. However, despite the use of a standard tool in Swaziland, there was no difference in the non-suppression rate amongst the re-testers which the authors associated with weaknesses in the counseling training and supervision [6]. Although WHO recommends adherence interventions for patients with detectable VL, no specification was given on the exact interventions to be undertaken [20]. Therefore, designing more efficient and effective follow up mechanisms for such patients is crucial. Further, it is critical to identify and answer key quality of care questions, such as: a) what happens when a VL result is received at a health facility?, b) do health care workers and patients understand the meaning and implication of different VL results?, c) are patients whose VL result is beyond the threshold given priority?, and d) are there any quality improvement interventions for such patients?

An elevated VL indicates poor adherence or resistance [21–25]. The current Uganda Ministry of Health viral load monitoring algorithm requires confirmatory testing after 6 months in the event of an elevated initial test [19]. This may lead to unnecessary delay in treatment switch for patients harboring resistance. Delaying ART switch for patients with resistance increases the risk of sexual transmission of ART-resistant strains [26–28], as well as the likelihood of subsequent failure on second-line therapy [21–24, 29] [30–32]. Therefore, the Ministry of Health should consider adopting resistance testing for persons with viral loads beyond the threshold or elevated viral loads. This will facilitate early identification of resistance and subsequent regimen switch.

We found that children and young adolescents were significantly more likely to be non-suppressed compared to the rest of the age groups. Similarly, a recent study conducted in the US reported that older patients were more likely to achieve viral suppression [33]. Treatment for children and adolescents presents several challenges including the complexity in ARV dosing and the need to adjust doses as the children grow, which may be difficult especially for providers who are not skilled enough with pediatric care or too busy to track the suppression status. Treatment for children may also be affected by the type of caretakers for the child (i.e. parents vs. other caretakers), schooling environment (for those in schools), and transition to adolescent [34, 35]. Stigma, fear of disclosure, and stress may affect younger people more than their older counterparts [36–38]. Keeping young people in care by using social workers, peer supporters, and training on pediatric disclosure among health workers has been reported to contribute to improved ART adherence and hence better virological outcomes [39]. Alcohol, recreational drug use, and low socioeconomic status also contribute to non-adherence and hence virological failure among the young people [40, 41].

Our findings reveal that virological non-suppression is a common phenomenon even among people with good adherence, which highlights the need to ensure wide coverage and access to viral load testing services. This requires huge investments in viral load laboratory testing infrastructure and services, including point of care diagnostics especially in hard to reach areas. Further, a sufficient pool of trained providers is key to ensuring proper tracking, timely viral loads and quick action (including adherence support) based on the results.

HIV-associated TB is common among people on ART in Africa [42]. A number of cross-sectional studies have highlighted a link between incident TB and virological non-suppression [43–46]. However, the causality may not be clear given the inherit weaknesses of cross-sectional studies. It is possible for virological failure to lead to incident TB.The risk of virological non-suppression may also be increased by concurrent ART and TB treatment majorly due to impaired treatment adherence and pharmacokinetics drug interactions. Patients on ART with active TB should thus be prioritised for viral load monitoring and adherence support. Strengthening; interventions that prevent incident TB during ART such as isoniazid prevent therapy and infection control at health facilities is crucial. Repeat testers after suspected failure with active TB registered the highest risk of non-suppression compared to TB patients amongst other sub-categories (for routine monitoring and suspected treatment failures). Repeat testers after suspected failure pending confirmation, are more likely not to adhere to their medicines and therefore being at highest risk for virological non-suppression compared to other sub-groups. In-addition, the 74% reported adherence level for these patients could partly explain this finding.

### Limitations

The major limitation with our study was use of program data which had missing records. To enhance data quality, the laboratory is attempting to reduce this gap by making immediate phone calls to facilities where the samples are derived from. The cross-sectional study design may have misclassified transient viraemia as ‘non-suppressor’ leading to higher proportions of patients with virological non-suppression. In-addition, the cross-sectional study design, could not allow causality to be established. Clustering due to repeated samples in the same individual may have influenced the results in either direction. However, because of inconsistencies in the numbering of samples, we were not able to identify any repeat testers; and for this reason, we were not able to account for clustering effect in the analysis. In addition, although many studies have identified patient’s CD4 count as one of the major factors associated with virological suppression, we did not assess it in our study. This was due to the fact that the data collection tool that was used to collect the data analyzed in this study did not have an option for patients’ current CD4 count. The other limitation is that there could have been selection bias arising from the fact that the samples with complete records could have been obtained from individuals who were more motivated to seek viral load testing; or that patients who were tested could have been more likely to be non-suppressors. However, given that we used secondary data, it is very difficult to tell the extent to which selection bias exists or affects the interpretation of results. Our overall non-suppression level of 11% (suppression of 89%) suggests that Uganda is close to achieving the 90% viral suppression which is the UNAIDS target among HIV infected individuals on treatment. However, our study sample was only 100, 678 HIV-positive individuals versus the 750,896 individuals who were on active ART by the end of 2014 [18]. Thus, our findings may not be fully representative of the entire population on ART in Uganda.

The strength of this study was its large sample size and data on samples derived at the national level and thus provides a national outlook on the critical challenge of non-suppression amongst different populations receiving ART. This study identifies populations that require enhanced and targeted interventions to optimize ART and raises questions for additional research in this field.

## Conclusion

Repeat testers after suspected failure registered the highest proportion of non-suppressed patients. Young age, poor adherence and having active TB increased the odds of virological non-suppression. Being on second/third line regimens was protective against non-suppression. We recommend close follow up and intensified targeted adherence support for suspected treatment failures, repeat testers after suspected failure, children and adolescents, and adherence to ART guidelines by ART clinics.

## Abbreviations

ART: Antiretroviral therapy
ARV: Antiretroviral
CD4: Cluster of differentiation 4
CI: Confidence interval
CPHL: Central Public Health Laboratories
DBS: Dry blood spots
ePMTCT: Prevention of Mother-To-Child Transmission
HCII: Health center two
HCIII: Health center three
HCIV: Health center four
HIV: Human immunodeficiency virus
HIV/AIDS: Human immunodeficiency virus/Acquired immunodeficiency syndrome
NRH: National Referral Hospital
OR_adj_: Adjusted odds ratio
OR_crude_: Crude odds ratio
RNA: Ribonucleic acid
RRH: Regional Referral Hospital
TB: Tuberculosis
UNAIDS: Joint United Nations Programme on HIV/AIDS
VL: Viral load
WHO: World Health Organization
